# Case Report: Prolonged survival in Schinzel–Giedion syndrome featuring megaureter and *de novo SETBP1* mutation

**DOI:** 10.3389/fped.2025.1534192

**Published:** 2025-03-07

**Authors:** Glenda M. Beaman, Benjamin W. Jarvis, Anju Goyal, David J. B. Keene, Max Cervellione, Filipa M. Lopes, Kay A. Metcalfe, Adrian S. Woolf, William G. Newman

**Affiliations:** ^1^ Division of Evolution, Infection and Genomics, The University of Manchester, Manchester, United Kingdom; ^2^ Manchester Centre for Genomic Medicine, St Mary’s Hospital, Manchester University NHS Foundation Trust (MFT), Manchester, United Kingdom; ^3^ Division of Cell Matrix Biology and Regenerative Medicine, Faculty of Biology, Medicine and Health, The University of Manchester, Manchester, United Kingdom; ^4^ Department of Paediatric Urology, Royal Manchester Children's Hospital, Manchester, United Kingdom

**Keywords:** gene, syndrome, ureter, *SETBP1*, megaureter, Schinzel–Giedion syndrome

## Abstract

**Background:**

Rare early-onset lower urinary tract (REOLUT) disorders affect the ureter, urinary bladder, or urethra and manifest before birth or in childhood. Monogenic causes have been reported in a subset of such individuals.

**Objectives:**

A possible genetic cause was considered in a child with a megaureter who had syndromic features.

**Subjects and methods:**

Whole-exome sequencing was undertaken in individuals with megaureter. Immunohistochemistry was performed in urinary tract tissues of unaffected human fetuses.

**Results:**

The index case presented at 6 months with urosepsis and was found to have a unilateral primary non-refluxing megaureter which required stenting of its distal portion. This, together with dysmorphic features and developmental delay, led to a clinical diagnosis of Schinzel–Giedion syndrome (SGS). She was found to carry a *de novo* missense variant in *SET binding protein 1* (*SETBP1*), c.2613T>G (GenBank: NM_015559.3) (p.Ile871Met), a gene previously implicated in SGS. She was in good general health at 11 years of age, an unusual outcome given that most individuals with SGS die in the first 2 years of life. SETBP1 was detected in the fetal urinary tract, both in the urothelium and in nerve trunks in the kidney hilum and around the ureter. No *SETBP1* gene variants were detected in eight further cases of megaureter.

**Conclusions:**

This case indicates the value of genetic testing when a REOLUT disorder is accompanied by syndromic signs outside the urinary tract. SETBP1 may drive the functional differentiation of the human fetal ureter.

## Introduction

Rare early-onset lower urinary tract (REOLUT) disorders affect the ureter, urinary bladder, or urethra and manifest before birth or in childhood (1). One such disease is primary non-refluxing megaureter (PMU) featuring ureteric dilatation of over 7 mm in the absence of vesicoureteric reflux (VUR), ureterocoele, or bladder outflow obstruction (BOO) (2). PMU accounts for up to 10% of cases of congenital hydronephrosis (2). It is sometimes associated with urinary flow impairment in the distal ureter when surgical interventions, such as reimplantation or stenting, have been recommended (3).

Some individuals with REOLUT disorders have been shown to carry variants of specific genes expressed in the developing LUT, consistent with monogenic etiologies (1). Here, we highlight an individual who presented with PMU in the context of a rare multisystem disorder called Schinzel–Giedion syndrome (SGS) (4, 5). SGS is characterized by global neurodevelopmental impairment leading to moderate-to-profound intellectual disability, epilepsy, hypotonia, spasticity, dysautonomia, hearing loss, and cerebral visual impairment (4). The affected individual was remarkable for her longevity compared with most other reported cases of SGS. We demonstrated that she carried a novel *de novo* pathogenic missense variant of *SET binding protein 1* (*SETBP1*), in a hotspot region of the gene where other variants have been reported in other individuals with SGS (4), and we excluded variants in other genes associated with REOLUT disorders. We additionally demonstrated, using immunohistochemistry, that SETBP1 is present in the human fetal ureter, consistent with the expectation that this molecule is needed for functional differentiation of the LUT.

## Subjects and methods

The index case and others with megaureter were recruited to a research study investigating genetic causes for REOLUT disorders. Informed consent was provided by the parents (Integrated Research Application System study code 286682) who were themselves asymptomatic. They also permitted the sharing of the facial image of the index case. Whole-exome sequencing (WES) was carried out for the index individual, as previously described by the Beijing Genomics Institute (BGI) (6). The paired-end sequencing was performed using a BGI exome kit version 4 (59M) 6G BGI-Seq500, and the expected average sequencing coverage was ≥50× per nucleotide. Sequencing reads were aligned to the human reference genome (GRCh37/Hg19) using the Burrows–Wheeler Aligner software (BWA-short v.0.6.2). Raw data (FASTQ files) were converted to Variant Call Format (VCF) and Binary Alignment Map (BAM) files, which are standard formats for storing variant data. Analysis of exome data was performed in-house using VarSeq v2.2 (Golden Helix, Inc., Bozeman, MT, USA; http://www.goldenhelix.com). Duplicate reads were removed. Initially, the genome data were filtered for rare or novel variants in genes previously associated with LUT. Subsequently, an agnostic approach to variant filtering was applied prioritizing variants with an ultrarare minor allele frequency for both dominant and recessive inheritance models and those with *in silico* evidence of pathogenicity.

For histology studies, first-trimester human tissues collected after maternal consent (Research Ethics Committee codes 08/H0906/21+5 and 18/NE/0290) were sourced from the Human Developmental Biology Resource (7). Immunohistochemistry of human fetal tissues was undertaken with an anti-SETBP1 antibody raised in rabbits (Proteintech, 16841-1-AP) using a general methodology for brightfield immunostaining as described (8). In detail, 5 μm sections were dewaxed and rehydrated before incubation for 20 min with 0.15% hydrogen peroxide solution (Sigma-Aldrich) to block endogenous peroxidase activity. Sections were boiled in sodium citrate buffer (10 mM) for 6 min for epitope retrieval prior to incubation overnight at 4°C, with an anti-SETBP1 antibody raised in rabbit (Proteintech, 16841-1-AP, 1:1,000/1:10,000) diluted in 3% goat serum. Sections were washed and incubated with goat anti-rabbit antibody (Abcam, ab6720) for 1 h at room temperature before incubation with DAB reagent (Vector Biolabs) for 2.5 min and counterstaining with hematoxylin. Sections were dehydrated, mounted, and observed microscopically.

## Results

**Figure 1:**
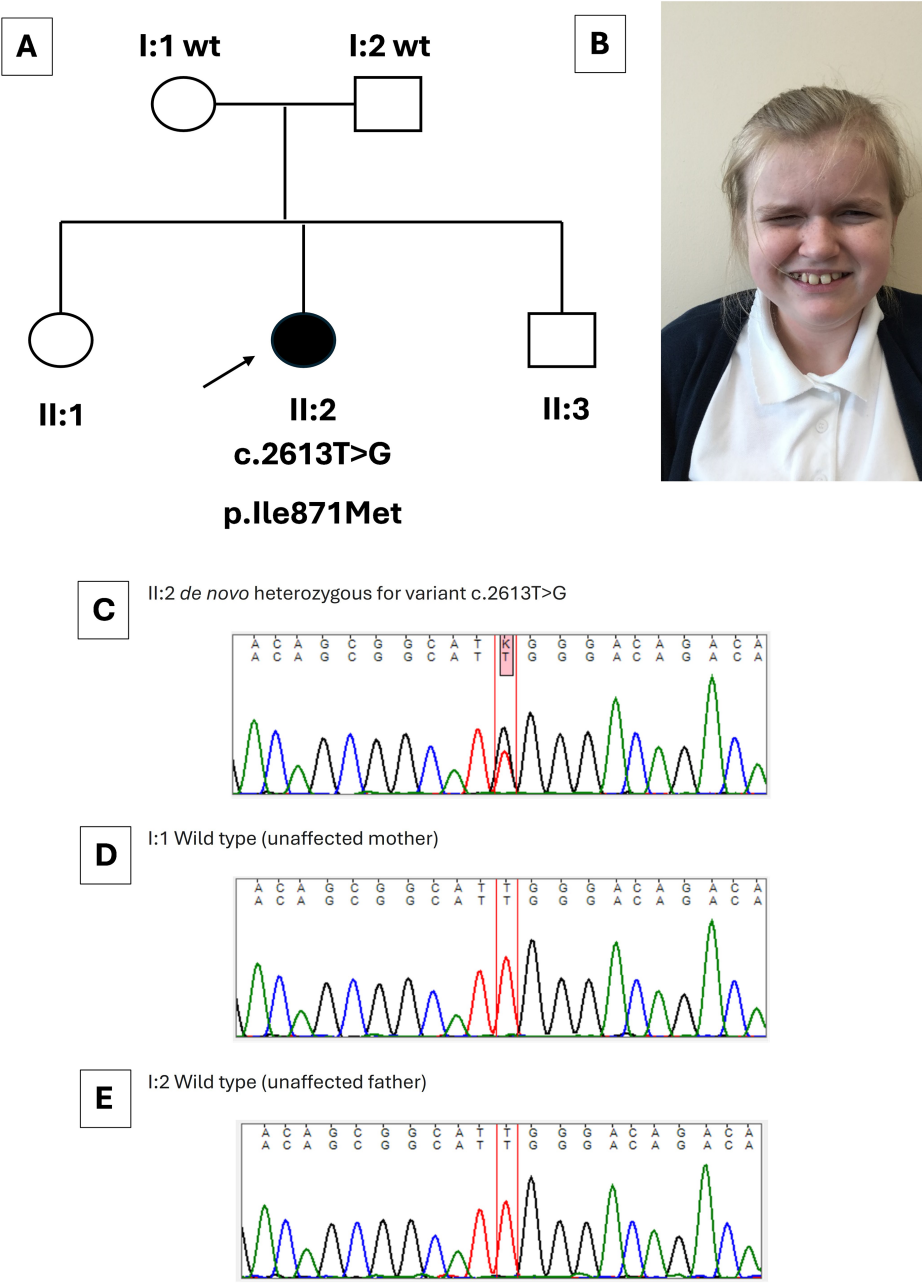
Clinical and genetic features of the index case. **(A)** Family pedigree, with an arrow indicating the index case (II:2) with her genotype shown directly below. Her healthy parents (I:1 and I:2) carried wild-type (wt) *SETBP1* sequence*.* The sister (II:1) and brother (II:3) of the index case were healthy. **(B)** Image of the index case at 12 years. Note characteristic features of Schinzel–Giedion syndrome including a prominent forehead, full cheeks, and infraorbital creases. **(C–E)** Sanger sequence traces. The index case **(C)** carried a *de novo* heterozygous *SETBP1* variant [c.2613T>G (GenBank: NM_015559.3) (p.Ile871Met)]. Normal sequences were detected in the healthy mother (I:1) and healthy father (I:2) **(D,E)**. The healthy siblings of the index case were not sequenced.

**Figure 2:**
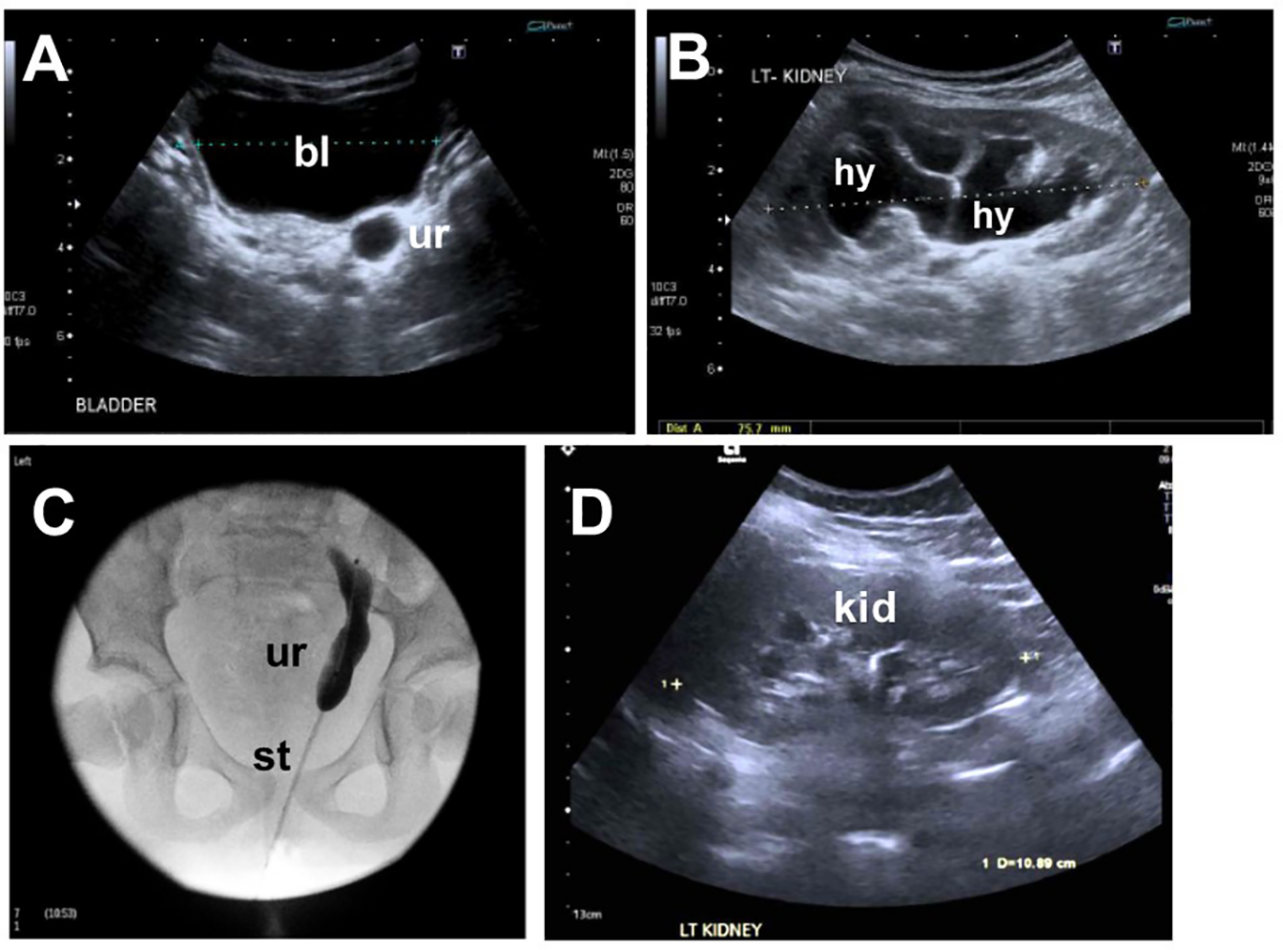
Imaging the lower urinary tract of the index case. **(A)** Ultrasound at 1 year of age showing a dilated distal ureter (ur) behind the bladder (bl). **(B)** Ultrasound at 1 year of age showing hydronephrosis (hy) of the left kidney. **(C)** Ureterogram at 4 years of age showing the stented megaureter. **(D)** Follow-up ultrasound shows resolution of hydronephrosis on the left kidney (kid).

The index case was a girl born to non-consanguineous parents of a White Irish background. She has two siblings who are fit and healthy (Figure 1A,B). Antenatal anomaly screening by ultrasonography (US) had been unremarkable. She was delivered at term weighing 3.88 kg (75th centile). At 6 months of age, recurrent febrile urinary tract infections (UTIs) prompted imaging investigations: US detected dilatation of 9.7 mm of the distal end of the left ureter (Figure 2A) and hydronephrosis of the left kidney (Figure 2B); micturating cystography showed a normal bladder and no VUR; and a mercaptoacetyltriglycine isotope renogram showed 47% function on the left. A diagnosis of PMU was made, and antibiotic prophylaxis was initiated. At 4 years of age, urosepsis recurred and US visualized debris in the pelvis of the left kidney. Overall kidney function was normal, with a plasma creatinine of 23 μmol/L. A 5 French balloon dilator was used to enlarge an abrupt narrowing at the distal end of the left ureter. A stent spanning the vesicoureteric junction was inserted (Figure 2C), which was removed 5 months later. Since then, hydronephrosis did not recur, and during her most recent urological follow-up, at 11 years of age, she had normal-sized kidneys (bipolar lengths: left, 10.9 cm; right, 11.1 cm) with normal appearing kidney parenchyma and no hydronephrosis (Figure 2D).

On general review at 11 years of age, she had normal somatic growth parameters: height, 152 cm (86th centile); weight, 53.4 kg (93rd centile); and head circumference, 53 cm (61st centile). She had several syndromic features outside the LUT. She had a narrow (reduced bitemporal diameter) head, with a prominent forehead, full cheeks, and narrow palpebral fissures (Figure 1B), and had curvatures of the second and fourth fingers of her left hand and fourth toes bilaterally. Her neurodevelopment was delayed, first speaking at 2 years and walking at 30 months. At 12 years old, she communicated using only a few words and was considered to have autistic spectrum disorder. She has experienced several febrile seizures and has a divergent squint of the right eye. She has reduced tear production, as previously described in children with SGS. There was no history of hearing loss, feeding problems, or abnormal bowel function.

A novel heterozygous variant in *SETBP1* (NM_015559.3):c.2613T>G p.(Ile871Met), leading to a missense change (p.Ile871Met) was identified in the index case. The variant was confirmed by Sanger sequencing, and testing her asymptomatic parents revealed that the variant had arisen *de novo* (Figure 1C–E). No variants were detected in other genes (*ACTG2*, *CHRM3*, *CHRNA3*, *EBF3*, *HPSE2*, *LRIG2*, *MYH11*, *MYL9*, *MYOCD*, *TBX18*, *TNXB*, *ROBO2*, *TSHZ3*, *UKP3A*) implicated in megaureter and other human REOLUT phenotypes (Table 1) (6, 9–20). Thus, based on clinical manifestations and genetic results, the index case had SGS. Given the *de novo* change in the index case and the fact that her siblings displayed no overt features of SGS, the sister and brother did not undergo gene sequencing. We also analyzed *SETBP1* in a megaureter cohort of eight index cases and found no pathogenic variants of this gene. Of these eight individuals, four had isolated megaureter with no other clinical features. In the other four, two were diagnosed with chromosomal anomalies partial tetrasomy of chromosome 22 (cat-eye syndrome) and trisomy 21 (Down syndrome). One individual had bilateral dysplastic kidneys, and another had severe epilepsy and developmental delay due to a variant in *FGF13*. The megaureter phenotype may be explained by the chromosomal anomalies in two of the additional cases (21).

**Table 1:** Some monogenic causes of megaureter and other human REOLUT phenotypes.

Gene abbreviation	Inheritance and OMIM disease/gene code	Encoded protein	LUT phenotype	Notable features outside the urinary tract	Reference
*ACTG2*	Autosomal dominant	Smooth muscle contractile protein	Megacystis	Microcolon	(9)
				Intestinal hypoperistalsis	
	619431				
*CHRM3*	Autosomal recessive	Acetylcholine receptor	Prune belly-like	Impaired pupillary light reflex	(10)
	100100				
*CHRNA3*	Autosomal recessive	Acetylcholine receptor	Functional BOO	Impaired pupillary light reflex	(11)
	118503				
*EBF3*	Autosomal dominant	Transcription factor	Functional BOO	Developmental delay, ataxia, facial dysmorphology	(6)
	617330				
*HPSE2*	Autosomal recessive	Antagonist of heparanase	Functional BOO	Grimace on smiling	(12)
	236730				
*LRIG2*	Autosomal recessive	Plasma membrane protein	Functional BOO	Grimace on smiling	(13)
	615112				
*MYH11*	Autosomal recessive	Smooth muscle contractile protein	Megacystis	Microcolon	(9)
				Intestinal hypoperistalsis	
	619351				
*MYL9*	Autosomal recessive	Smooth muscle cytoplasmic protein	Megacystis	Microcolon	(14)
				Intestinal hypoperistalsis	
	619365				
*MYOCD*	Autosomal dominant	Transcription-associated factor	Prune belly syndrome that is sex (male)-limited	Congenital heart defects	(15)
	618719				
*ROBO2*	Autosomal dominant	Plasma membrane receptor	Megaureter, VUR	-	(16)
	610878				
*SETBP1*	Autosomal dominant	DNA-binding protein	Megaureter	Severe developmental delay	4 and this report
	269150				
*TBX18*	Autosomal dominant	Transcription factor	Megaureter	-	(17)
	143400				
*TNXB*	Autosomal dominant	Extracellular matrix protein	VUR, duplex ureter	Hypermobility	(18)
	615963				
*TSHZ3*	Autosomal dominant	Transcription factor	Hydronephrosis	Autism spectrum	(19)
	614119				
*UPK3A*	Autosomal dominant	Urothelial coating protein	VUR, kidney dysplasia	-	(20)
	611559				

Online Mendelian Inheritance in Man (OMIM) disease/gene code numbers are listed in addition to the gene abbreviation, the mode of inheritance, LUT phenotype, and other key features external to the LUT.

**Figure 3:**
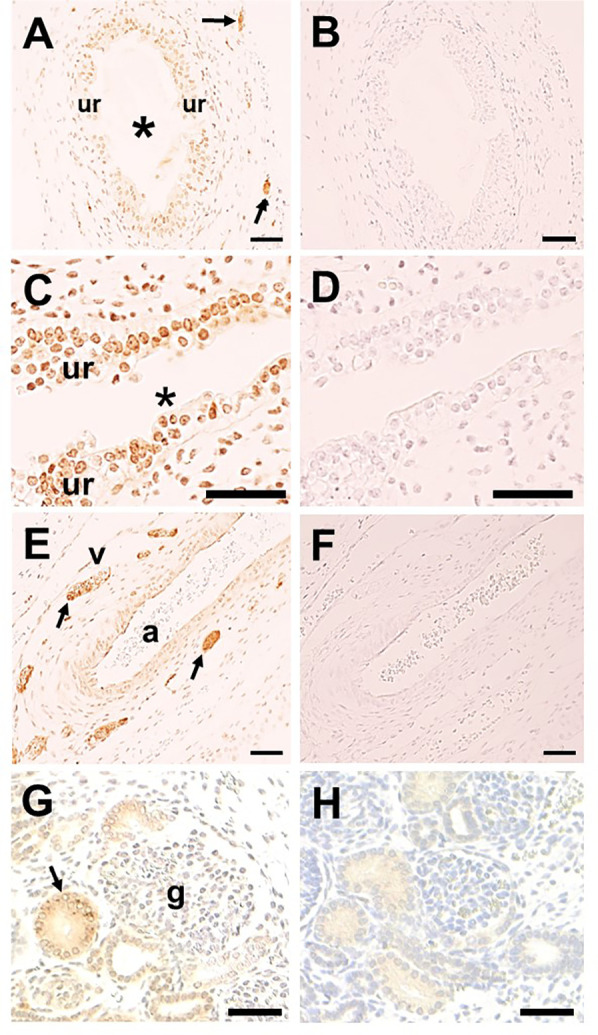
SETBP1 immunohistochemistry. Representative images from human fetal urinary tracts at 10 and 12 weeks of gestation. All sections counterstained with hematoxylin (blue nuclei). **A**, **C**, **E**, and **G** were probed with the SETBP1 antibody (1:1,000 in **A**, **C**, and **E**; 1:10,000 in **G**), while **B**, **D**, **F**, and **H** are nearby sections with the primary antibody omitted. Positive immunostaining appears brown. SETBP1 was detected in urothelial cells in both the ureter (ur in **A**; asterisk indicates the lumen) and the kidney pelvis (ur in **C**; asterisk indicates the lumen). Stomal cells in the kidney pelvis were also positive **(C)**. Putative nerve trunks immunostained for SETBP1 in loose tissues around the ureter (arrows in **A**) and in the neurovascular bundle at the kidney hilum (arrows in **E**; a indicates artery, and v indicates vein). Within the kidney, SETBP1 was detected in a subset of tubules (arrow in **G**) while glomeruli (g in **G**) were negative. Note that no significant signals were detected in the negative control sections **(B,D,F,H)**. Scale bar: 50 μm in all frames.

SETBP1 immunohistochemistry was undertaken in LUTs of two human fetuses of 10 and 12 weeks gestation when ureters contain differentiating smooth muscle surrounding a urothelial tube (8), while the metanephric kidney contains the first layers of nephrons together with branching collecting ducts converging on the kidney pelvis (22). In the ureter itself, SETBP1 was detected in the urothelium and putative nerve trunks in interstitial tissues adjacent to the organ (Figure 3A,B). Moreover, urothelia in the kidney pelvis was also immunostained for SETBP1 (Figure 3C,D). In the neurovascular bundle entering the hilum of the fetal kidney, prominent SETBP1 immunostaining was detected in nerve trunks, but neither the artery nor vein was immunostained (Figure 3E,F). Within the fetal metanephric kidney, a subset of cortical tubules immunostained for SETBP1 but glomeruli were negative (Figure 3G,H).

## Discussion

The constellation of clinical features constituting SGS was first described in 1978 (23), with emphases on “midface hypoplasia, congenital heart defect, and hydronephrosis.” Since then, approximately 90 individuals with SGS have been reported (4). Up to half of individuals with this syndrome die by 2 years of age, usually from respiratory failure/pneumonia or epilepsy (24). Apart from a report of an individual with SGS alive at 15 years (25), there are no published reports of affected individuals surviving into the second decade of life. Thus, the longer survival of the current index case, still in good general health at 11 years apart from neurodevelopmental delay, is remarkable.

LUT disease is a key feature of SGS, with 31 of 35 cases having hydronephrosis associated with stenosis at the proximal or distal end of the ureter or having VUR (24). Hydronephrosis in SGS can be detected before birth, but this is not invariable (24), as was the case in the current individual. In contrast to the LUT, primary disease of the kidney is usually absent although a small subset of SGS patients have kidney cysts (4). In one extraordinary individual with SGS, a multidysplastic kidney was reported which gave rise to malignancy (26). While LUT disease is an integral part of SGS, conversely this syndrome is an unusual cause of REOLUT. For example, of 143 cases of congenital ureteropelvic obstruction, just one individual with SGS was noted (27). This concurs with our current study where pathogenic *SETBP1* variants were absent in eight patients with megaureter.

The association of SGS with pathogenic variants in *SETBP1* was first reported in 2010 (28). Subsequently, gene testing has shown that the majority of, but not every (29), individual with clinical features of this syndrome carries a heterozygous missense variant in *SETBP1* (4). As with the current case, reported *SETBP1* variants arise *de novo* (4). Moreover, again as in the current individual, most genetically defined reported mutations affect amino acids 868–871 where they are considered to confer novel properties on the encoded protein so that it acts in a “dominant-negative” or “gain-of-function” manner (4, 28). Of note, variants at residue 871 (both p.Ile871Ser and Ile871Thr) have previously been reported in individuals with SGS (4). However, the specific Ile871Met variant identified in our patient has not been previously reported. This novel variant may account for why our patient has lived longer and does not have all of the classical features of SGS previously reported, including a short nose and overt midface retraction, microcephaly, a protruding tongue, hearing loss, or epilepsy. However, such variability in ultrarare neurodevelopmental disorders is recognized due to previous genetic testing being confined to individuals only with all of the classical features. In addition, some facial characteristics change with age and so are less likely to have been described in older children where overall survival is reduced.

The protein encoded by the *SETBP1* gene binds DNA where it can activate a program of gene expression through the recruitment of a set of protein partners (HCF1/KMT2A/PHF8) (30). Experimental expression of mutated *SETBP1* in developing mouse brains causes impaired neurogenesis (30). Given that LUT malformations are an integral part of SGS, as in the current report, we asked whether SETBP1 protein could be detected in the developing human LUT. Using immunohistochemistry, SETBP1 was detected in various cell types, including the urothelium and nerve bundles. Mouse experiments have shown that the embryonic urothelium acts as a paracrine signaling center, enhancing the differentiation of smooth muscle cells in the ureter wall (31). Furthermore, resected segments of human PMUs, not associated with SGS, display a thin smooth muscle layer and sparse interstitial cells of Cajal (32), neural-like cells located in the smooth muscle layer. We hypothesize that *SETBP1* mutations perturb the generation of peristaltic waves that propel urine to the bladder (33) by impairing the differentiation of ureteric smooth muscle and innervation, thus leading to megaureter. Our detection of SETBP1 protein in a subset of human fetal kidney tubules accords with a mouse study (34) and the pattern reported in the adult human kidney (35). Future clinical studies will be needed to determine whether defects of tubule function exist in individuals with SGS.

## Conclusions

SGS is a rare cause of LUT disorders. It should be suspected in individuals with hydronephrosis or megaureter with features consistent with the SGS characteristic facial appearance and neurodevelopmental delay. Our study strengthens the value of genetic testing when a REOLUT disorder is accompanied by clinical signs outside the urinary tract. SETBP1 may drive the functional differentiation of the human fetal ureter.

## Data Availability

The raw data supporting the conclusions of this article will be made available by the authors, without undue reservation.
